# Implications of HIF-1α in the tumorigenesis and progression of pancreatic cancer

**DOI:** 10.1186/s12935-020-01370-0

**Published:** 2020-06-24

**Authors:** Xiao Jin, Lu Dai, Yilan Ma, Jiayan Wang, Zheng Liu

**Affiliations:** Medical Center for Digestive Diseases, Second Affiliated Hospital, Nanjing Medical University, 121 Jiangjiayuan Road, Nanjing, 210011 Jiangsu China

**Keywords:** Hypoxia, HIF-1α, Pancreatic cancer, Tumorigenesis, Progression, Review

## Abstract

Pancreatic cancer is one of the leading causes of cancer-related deaths worldwide and is characterized by highly hypoxic tumor microenvironment. Hypoxia-inducible factor-1 alpha (HIF-1α) is a major regulator of cellular response to changes in oxygen concentration, supporting the adaptation of tumor cells to hypoxia in an oxygen-deficient tumor microenvironment. Numerous studies revealed the central role of HIF-1α in the carcinogenesis and progression of pancreatic cancer. This article reviewed the molecular mechanisms of how HIF-1α regulated tumorigenesis and progression of pancreatic cancer and suggested that targeting HIF-1α and its signaling pathways could be promising therapeutics for pancreatic cancer.

## Background

According to the latest global cancer statistics in 2018, pancreatic cancer accounts for 2.5% of new cancers worldwide, and mortality accounts for 4.5% of all cancer deaths [1]. An assessment of tumor morbidity and mortality expects pancreatic cancer to rise to the second highest cancer mortality in the United States by 2030 [2]. At present, the high malignancy and poor curative effect of pancreatic cancer are mostly attributed to hypoxic tumor microenvironment [3, 4].

**Fig. 1:**
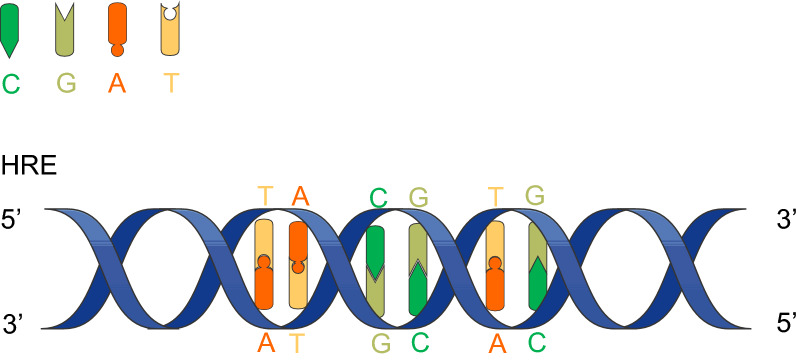
HRE core sequence

**Fig. 2:**
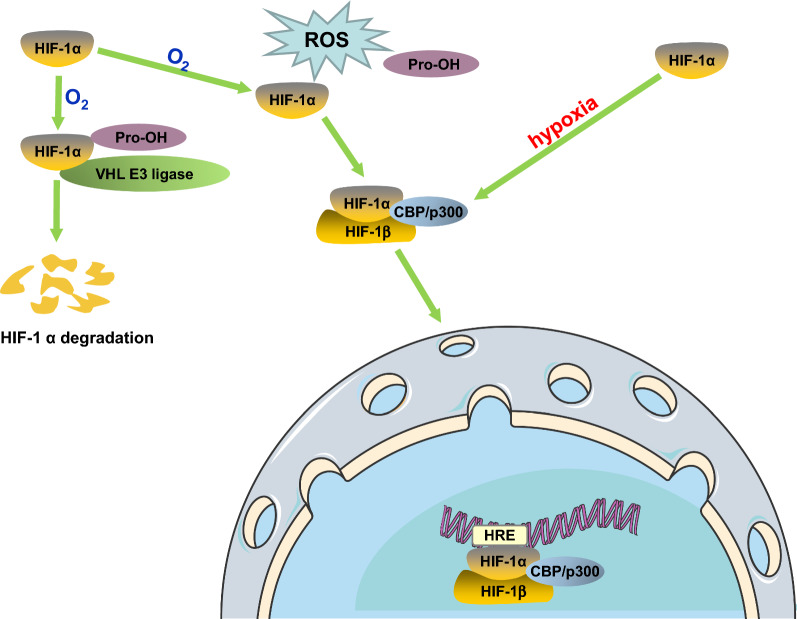
HIF-1α degradation and activation. ↑, promote. Under normoxia, HIF-1α is hydroxylated by prolyl hydroxylases and binds to VHL which recruits E3-ubiquitin ligase to interact with HIF-1α, resulting in degradation of HIF-1α in a ubiquitin–proteasome way. Besides, the existence of ROS in normoxia inhibits the acetylation of HIF-1α via blocking the activation of PHDs, protecting HIF-1α from degradation. In hypoxia, oxygen deficiency inhibits hydroxylation of HIF-1α, HIF-1α forms heterodimers with HIF-1β with help of CBP/p300 and transfers to the nucleus to bind to its target genes

Hypoxia-inducible factor-1 (HIF-1) is a key factor regulating cell adaptation to hypoxia [5]. HIF-1 consists of an oxygen-regulated alpha subunit (HIF-1α) and a constitutively expressed beta subunit (HIF-1β) [6]. Under normoxic conditions, the proline and lysine residues on the oxygen-dependent degradation domain of HIF-1α are hydroxylated, and the modified HIF-1α interacts with the Von Hippel–Lindau E3 ubiquitin ligase complex followed degradation through the ubiquitin–proteasome pathway [7]. However, HIF-1α is stable in hypoxia and forms heterodimers with HIF-1β with the help of coactivators such as cyclic adenosine monophosphate response element-binding protein (CBP) and acetyltransferase (p300), and then, HIF-1α transfers to the nucleus and binds to the target gene hypoxia response element (HRE), a DNA sequence consisting of consecutive transcription factor binding sites that contains the core sequence of 5′-TACGTG-3′ (Fig. 1), modulating the targets transcription [5, 8]. In addition, HIF-1α can also be activated by an oxygen-independent mechanism [9] (Fig. 2).

Pancreatic cancer possesses hypoxic niche and is accompanied by HIF-1α overexpression [10, 11]. Increasing studies explore the roles of HIF-1α in pancreatic cancer and pancreas embryonic development. This review mainly elucidated the major function of HIF-1α in the carcinogenesis and progression of pancreatic cancer as well as pancreas embryonic development. Therefore, targeting HIF-1α and its signaling pathways might be effective therapeutic approaches for pancreatic cancer.

### HIF-1α in pancreas embryonic development and homeostasis

Pancreatic blood flow is low and cells are hypoxic during the early stages of embryogenesis. Later, increasing oxygen concentration facilitates pancreatic cells further differentiation [12]. HIF-1α level gradually decreases and plays a central role in responding to changes in oxygen during pancreas embryonic development [13]. Low expression of HIF-1α in islets regulated glucose-stimulated insulin secretion and protected β-cells reserve and function via binding to HRE in the promoter of aryl hydrocarbon receptor nuclear translocator (ARNT), but deletion of HIF-1α impaired β-cells function [14]. Congruously, appropriate level of HIF-1α-mediated vascular endothelial growth factor A (VEGF-A) expression contributed to normal pancreatic growth and development of islet-specific capillary fenestrations, maintaining islet β-cells mass and function [15]. However, high expression of HIF-1α lost the function of maintaining pancreas development and its homeostasis. Studies suggested overexpressed HIF-1α inhibited the differentiation of pancreatic endocrine progenitor cells via suppressing the expression of neurogenin3, a pro-endocrine transcription factor, through activating mTOR complex I signaling, and suppressed islet β-cells differentiation via mediating hypervascularization [13, 16, 17]. Besides, data indicated diabetes and higher blood glucose levels among those without diabetes were potential risks of pancreatic cancer, hyperglycemiainduced HIF-1α overexpression and microenvironment hypoxia, upregulating MMP-9 expression and promoting pancreatic cancer progression in a HIF-1α-dependent manner. Hyperglycemia was mostly attributed to β-cells dysfunction, while low level of HIF-1α was required for β-cells function maintenance. This might imply that the different roles of HIF-1α in development and carcinogenesis of pancreas depended on the difference in its expression levels [18, 19]. Surprisingly, upregulation of HIF-1α does not necessarily exert pathogenic or carcinogenic effects in pancreas. HIF-1α acted as a downstream molecule of mTOR and regulated glucagon-like peptide-1 (GLP-1) receptor-induced metabolism reprogramming via PI3K/mTOR pathway, enhancing mice islet viability [20, 21]. Moreover, HIF-1α accumulation contributed to pancreas tissue regeneration via inhibiting intrapancreatic B lymphocytes accumulation in cerulein-induced experimental mice pancreatitis [22]. In addition, islets cultured in vitro tended to lose their heavy vascularization, but hypoxia-induced HIF-1α could maintain this vasculature via enhancing vascular length and endothelial cells area through upregulating VEGF-A expression, facilitating transplantation survival [23]. Additionally, the protective effect of exendin-4, a GLP-1 receptor agonist, for transplantation islets during hypoxic phase was also attributed to overexpression of HIF-1α [24]. However, current researches have consistently revealed that HIF-1α is overexpressed in patients with pancreatic cancer and regulated various genes expression (Table 1), acting as an oncogene in pancreatic cancer [10, 11].

**Table 1:** Genes induced by HIF-1α in pancreatic cancer tumorigenesis and progression

Target genes	Acting mechanisms	References
LncRNA-NUTF2P3-001	↑LncRNA-NUTF2P3-001, ↓miR-3923, ↑KRAS, ↑cells viability, proliferation and invasion	[26]
LncRNA-CF129	↓LncRNA-CF129, ↑p53, ↑FOXC2, ↑cells proliferation	[28]
STIM1	↑STIM1, ↑cells proliferation, invasion and anchorage independent growth	[10]
PKM2	↑PKM2, ↑glycolysis	[31]
ENO1, PGM2	↑ENO1 and PGM2, ↑glycolysis	[33]
GLUT1, LDHA, HK2	↑GLUT1, LDHA and HK2, ↑glycolysis	[37]
CypA	↑CypA, ↓apoptosis, ↑cells proliferation, migration and invasion	[38]
MiR-21	↑MiR-21, ↓apoptosis, ↑cells proliferation	[40]
MT2-MMP	↑MT2-MMP, ↓apoptosis, ↑cells proliferation and invasion	[42]
MTA2	↑MTA2, ↓E-cadherin, ↑EMT, cells migration and invasion	[49]
Twist	↑Twist, ↓ E-cadherin, ↑EMT and cells proliferation	[50]
Snail	↑Snail, ↓E-cadherin, ↑N-cadherin, ↑EMT, cells migration and invasion	[51]
LncRNA-BX111	↑LncRNA-BX111, ↑ZEB1, MMP-2, ↓E-cadherin, ↑EMT, ↑tumor growth and metastasis	[52]
CD133	↑CD133, ↑tumor stem cell properties, ↑cells migration and invasion	[56]
RER1	↑RER1, ↑N-cadherin, Vimentin and Snail, ↓E-cadherin, ↑Sox2, Bmi1, Lin28 and Nanog, ↑EMT and cancer stem cell-like properties	[58]
ATG5, Beclin1	↑ATG5 and Beclin1, ↑autophagy and cancer stem cell-like properties	[59]
ABCG2	↑ABCG2, ↑ chemoresistance	[64]
NF-κB	↑NF-κB, ↓E-cadherin, ↑N-cadherin, Vimentin, Snail, Twist, ↑EMT and chemoresistance	[65]
CXCR4	↑CXCR4, ↑chemoresistance	[66]
IL-37	↓IL-37, ↑chemoresistance	[68]
VEGF-A	↑VEGF-A, ↑angiogenesis, ↑tumor growth	[73]
STAT3, VEGF-A	↑VEGF-A and STAT3, ↑angiogenesis, ↑tumor growth	[75]
CHC, VEGF-A	↑CHC and VEGF-A, ↑angiogenesis, ↑tumor growth	[77]
VEGF, MMP-2, MMP-9	↑VEGF, MMP-2 and MMP-9, ↑angiogenesis, ↑tumor growth	[78]
ADAM10	↑AMAD10, ↓mMICA, ↑sMICA, ↓NKG2D, ↓NK cells cytotoxicity, ↑immune evasion	[82]
CCL2	↑CCL2, ↑α-SMA, ↑desmoplasia	[85]
SHH	↑SHH, ↑HH signaling, ↑ collagen Ι and fibronectin, ↑desmoplasia	[84, 86]

↑, promote; ↓, inhibit; KRAS, V-Ki-ras2 Kirsten Rat Sarcoma Viral Oncogene Homolog; FOXC2, forkhead box C2; STIM1, stromal interaction molecule 1; PKM2, M2 isoform of pyruvate kinase; ENO1, enolase 1; PGM2, phosphoglucomutase-2; GLUT1, glucose transporter type 1; LDHA, lactate dehydrogenase A; HK2, hexokinase 2; CypA, cyclophilin A; MT2-MMP, membrane type-2 matrix metalloproteinase; MTA2, metastasis-associated protein 2; EMT, epithelial mesenchymal transition; ZEB1, zinc finger E-box-binding protein 1; MMP-2, matrix metalloproteinase 2; RER1, retention in endoplasmic reticulum 1; ATG5, autophagy related 5; ABCG2, ATP-binding cassette subfamily G member 2; CXCR4, chemokine receptor 4; VEGF-A, vascular endothelial growth factor A; STAT3, signal transducer and activator of transcription 3; CHC, clathrin heavy chain; MMP-9: matrix metalloproteinase 9; ADAM10, a disintegrin and metalloproteinase domain 10; mMICA, membrane major histocompatibility complex class I molecular associated proteins A; sMICA, soluble major histocompatibility complex class I molecular associated proteins A; NKG2D, natural killer group 2 member D; CCL2, chemical chemokine 2; α-SMA, α-smooth muscle actin; SHH, sonic hedgehog; HH, hedgehog

### HIF-1α and pancreatic cancer parenchyma

#### Survival and proliferation

Aberrant cells proliferation is the most basic feature in the tumorigenesis. Studies indicate HIF-1α promotes the proliferation of pancreatic cancer cells through various mechanisms. lncRNA is involved in the modulation of HIF-1α in the carcinogenesis [25]. In pancreatic cancer, HIF-1α bound to the HRE of lncRNA-NUTF2P3-001, upregulating its expression. Overexpressed lncRNA-NUTF2P3-001 competitively bound to miR-3923, increasing V-Ki-ras2 Kirsten Rat Sarcoma Viral Oncogene Homolog (KRAS) expression, which resulted in significant improvement of tumor cells survival rate and proliferation [26]. Besides, lncRNA-FEZF1-AS1 enhanced pancreatic cancer cells proliferation via the miR-142/HIF-1α axis in hypoxia [27]. Moreover, decreased IncRNA-CF129 regulated by HIF-1α/histone deacetylase 1(HDAC1) complex facilitated pancreatic cancer progression via increasing forkhead box C2 (FOXC2) expression, and FOXC2 overexpression was induced by E3 ligase MKRN1-mediated p53 ubiquitin degradation. Furthermore, FOXC2 and HIF-1α regulated reciprocally and formed a positive feedback loop [28]. Stromal interaction molecule 1 (STIM1) is regulated by HIF-1α and is implicated in the cells proliferation. The study indicated STIM1 expression was upregulated, accompanied by HIF-1α overexpression in pancreatic cancer tissues. HIF-1α bound to the HRE of STIM1 and elevated its expression, thereby increasing the proliferation of pancreatic cancer cells [10].

#### Metabolism reprogramming

High metabolism is a notable property of tumor cells. Aerobic glycolysis pathway can produce large amounts of adenosine triphosphate in a short period of time and is one of the most important metabolic modes of tumor cells [29]. HIF-1α is considered as a primary conditioner of metabolism reprogramming [30]. Decreased Rho GTPase-activating protein 4 (ARHGAP4) facilitates aerobic glycolysis of pancreatic cancer through activating HIF-1α pathway and upregulating M2 isoform of pyruvate kinase (PKM2) expression [31]. Mucin1 is a type I transmembrane protein which is widely expressed in pancreatic cancer tissues and regulates anabolic glucose metabolism in a HIF-1α-dependent manner in pancreatic cancer [32]. Mucin1 enhanced the stability of HIF-1α and recruited HIF-1α and p300 to bind to the promoter of glycolytic genes such as enolase1 (ENO1) and phosphoglucomutase-2 (PGM2), upregulating their expression which contributed to increased glucose uptake and lactate production in pancreatic cancer cells [33]. Moreover, mucin1-mediated HIF-1α stability enhanced gemcitabine resistance via increased glucose metabolism [34]. Similarly, several studies confirmed that HIF-1α increased expression of glycolytic-related enzymes and the production of lactic acid, meeting the metabolic needs of pancreatic cancer cells [34, 35]. Besides, the transcription factor 7-like 2 (TCF7L2) is correlated with the glycolysis in tumor cells [36]. The research indicated upregulation of TCF7L2 inhibited the promoter activity of Egl-9 family hypoxia-inducible factor 2 (EGLN2) and suppressed its expression, which enhanced the stability of HIF-1α, enhancing glycolysis-related genes expression and increasing glycolysis in pancreatic cancer cells [37].

#### Anti-apoptosis and autophagy

Numbers of studies revealed high expression of HIF-1α markedly enhanced the anti-apoptotic capacity of pancreatic cancer cells [34, 35]. In addition, research showed HIF-1α bound directly to the HRE of cyclophilin A (CypA) and upregulated its expression, which inhibited pancreatic cancer cells apoptosis [38]. Besides, miR-21 is associated with tumor cells evading apoptosis [39]. In pancreatic cancer, HIF-1a induced miR-21 overexpression, preventing tumor cells from apoptosis in an oxygen-deficient environment [40]. Membrane type-2 matrix metalloproteinase (MT2-MMP), one of the members of the matrix metalloproteinase family, is expressed in tumor cells and is implicated in proliferation, migration and invasion of them [41]. Recent researches showed MT2-MMP was a new component in an anti-apoptotic pathway network in tumor cells and was also a novel target of HIF-1α. HIF-1α bound to the HRE of MT2-MMP and activated its transcription, overexpressing MT2-MMP clearly mitigated the apoptosis of pancreatic cancer cells [42]. Nevertheless, the roles of HIF-1α in the apoptosis of pancreatic cancer cells remain controversial. Dai et al. reported that HIF-1α could induce cells apoptosis in pancreatic cancer [43]. Therefore, the involvement of HIF-1α in the regulation of apoptosis in pancreatic cancer in hypoxia requires further exploration.

It is well known that hypoxia-induced autophagy could promote tumor progression [44]. Autophagy is a metabolic pathway in which cells transport their proteins and organelles to lysosomal degradation, reducing their oxidative stress [45]. The study indicated that HIF-1α-induced autophagy potentiated epithelial mesenchymal transition (EMT) and migration of pancreatic cancer stem cells, increasing the malignancy of pancreatic cancer [46]. Besides, HIF-1α-mediated autophagy reduced lumican level secreted by pancreatic stellate cells, promoting pancreatic cancer progression [47].

#### EMT, invasion and metastasis

A large number of studies demonstrated HIF-1α-mediated pancreatic cancer cells EMT, invasion and metastasis in hypoxia. Current researches focus on HIF-1α regulating EMT processes via affecting EMT-related proteins expression. Epithelial cell cadherin (E-cadherin) is a single transmembrane glycoprotein encoded by the cadherin1 gene that maintains epithelial cell polarity and cell-to-cell contact. Deletion of E-cadherin induces EMT, relating to invasion and metastasis in cancers [48]. HIF-1α could inhibit E-cadherin expression via recruiting metastasis-associated protein 2 (MTA2)/HDAC1 complex to bind to E-cadherin promoter, inducing EMT in pancreatic cancer cells [49]. Besides, the transcription of E-cadherin is also regulated by Twist, which is regulated by HIF-1α in hypoxia and serves as an important transcription factor promoting EMT of tumor cells. In pancreatic cancer, Twist recruited Ring1B and enhancer of zeste homolog 2 (EZH2), members of polycomb family, to bind to the promoter of E-cadherin and suppressed its transcription, inducing EMT [50]. Moreover, HIF-1α increased Snail transcription through binding to its HRE, contributing to EMT in pancreatic cancer [51]. Furthermore, numerous studies showed lncRNAs were involved in the regulation of EMT. Deng et al. reported that HIF-1α and lncRNA-BX111887 were overexpressed in pancreatic cancer tissues. Under the mediation of HIF-1α, lncRNA-BX111887 recruited transcriptional factor Y-box protein to the promoter of zinc finger E-box-binding protein 1 (ZEB1), a major factor inducing EMT, and promoted its transcription [52]. Additionally, miRNAs are implicated in the regulation of HIF-1α on the EMT of pancreatic cancer cells. MiR-142 expression in pancreatic cancer tissues and pancreatic cancer cell lines was significantly lower than that in normal tissues. Down-regulated miR-142 increased the expression of HIF-1α, upregulating EMT-related proteins, enhancing the invasion and migration of pancreatic cancer cells [53].

#### Tumor stem cells

It is well known that tumor stem cells are the key to tumor cells clone formation, proliferation and migration, which are not easily eliminated by anti-tumor drugs and can further differentiate as well as support tumor development [54]. One of the reasons for pancreatic cancer recrudescence and treatment resistance is the presence of tumor stem cells [55]. CD133 is one of surface molecules of pancreatic cancer stem cells, and its expression is increased in a HIF-1α-dependent manner in hypoxia [56]. Moreover, CD133 elevated HIF-1α transcriptional activity in pancreatic cancer cells in hypoxia, initiating its expression and activating its target genes, which in turn induced EMT phenotype and tumor cells migration [57]. Besides, the study indicated HIF-1α bound to the regulatory region in the upstream of the initiation codon of retention in endoplasmic reticulum 1 (RER1) and increased its transcriptional activity, enhancing pancreatic cancer stem cells properties and inducing EMT [58]. Additionally, the study elucidated that HIF-1α-induced autophagy mediated the conversion of non-stem pancreatic cancer cells to pancreatic cancer stem cells in hypoxia, enhancing the malignancy of pancreatic cancer [59].

#### Chemoresistance and radiotherapy resistance

Most pancreatic cancer patients are diagnosed in the advanced stages or even with distant metastasis due to absence of early recognizable symptoms, missing the opportunity for radical surgical resection. Thus, chemotherapy and radiotherapy have become the main therapies for pancreatic cancers currently, but the efficacy of chemotherapy and radiotherapy are limited by factors inside and outside the tumor cells [60, 61]. The study suggested hypoxia-induced HIF-1α contributed to chemoresistance and radiotherapy resistance in pancreatic cancer [62]. ATP-binding cassette subfamily G member 2 (ABCG2) is a multidrug resistant pump that is associated with drug resistance in numerous malignancies [63]. In pancreatic cancer, hypoxia-induced phosphorylation of ERK1/2 activated HIF-1α, increasing the accumulation of HIF-1α in the cytoplasm and translocating to the nucleus, and then bound to the HRE of ABCG2 and promoted its transcription, thereby enhancing the drug resistance [64]. Several studies revealed that HIF-1α and NF-κB expression were increased in pancreatic cancer tissues and there was a positive feedback regulation between them, resulting in chemoresistance partly. In pancreatic cancer, gemcitabine treatment activated NF-κB and HIF-1α via ROS-mediated activation of ERK1/2 and Akt, upregulating chemokine receptor 4 (CXCR4) expression and contributing to gemcitabine resistance by CXCR4/chemokine 12 (CXCL12) signaling [65, 66]. Besides, hypoxia-induced miR-301a overexpression induced gemcitabine resistance via enhancing HIF-1α accumulation through suppressing expression of TAp63 which could down-regulate HIF-1α expression through proteasomal degradation [67]. IL-37 expression was decreased in pancreatic cancer, down-regulated IL-37-mediated gemcitabine resistance via interacting with HIF-1α and signal transducer and activator of transcription 3 (STAT3) and forming the HIF-1α/IL-37/STAT3 negative feedback [68]. Heat shock protein90 (HSP90) is a pivotal molecular chaperone of HIF-1α, which plays a crucial role in the correct folding, stability and transcription of HIF-1α. Inhibiting HSP90 could reverse HIF-1α-mediated resistance to radiotherapy and chemotherapy in pancreatic cancer [69, 70].

### HIF-1α and pancreatic cancer stroma

#### Angiogenesis

Tumor angiogenesis is known to be essential for growth and metastasis of pancreatic cancer [71]. VEGF regulated by HIF-1α at transcription level is essential to angiogenesis [72]. Azoitei et al. reported VEGF-A was not only a target of HIF-1α, but also of NF-κB transcription factor, involved in regulating tumor progression. In pancreatic cancer, PKM2 upregulated HIF-1α in a NF-κB/p65-dependent manner, inducing VEGF-A expression which contributed to tumor angiogenesis and growth [73]. The study showed both STAT3 and HIF-1α were client proteins of HSP90 [74]. In pancreatic cancer, HSP90 promoted VEGF-mediated angiogenesis via activating IL-6/HIF-1α/STAT3 autocrine loop [75]. Serine/threonine kinase 33 (STK33), a serine/threonine kinase, is a new client protein of HSP90. STK33 participated in tumor angiogenesis promoted by HSP90 chaperone via upregulating HIF-1α accumulation and its target gene VEGF secretion in pancreatic cancer [76]. Clathrin is a trimer of heavy chains, each paired with a light chain. Clathrin heavy chain (CHC) interacted with HIF-1α and co-localized to the HRE in the VEGF-A promoter region, upregulating VEGF-A expression to increase angiogenesis in pancreatic cancer [77]. Intriguingly, HIF-1α could also mediate stress-induced pancreatic tumor growth and angiogenesis via regulating the expression of VEGF, matrix metalloproteinase 2 (MMP-2) and matrix metalloproteinase 9 (MMP-9) [78].

#### Immune evasion

The surfaces of tumor cells generally express new antigens, which are recognized by immune system, subsequently activating innate and acquired immune responses. However, tumor cells could evade immune responses via modifying the surface antigens or altering the tumor microenvironment [79]. Major histocompatibility complex class I molecular-associated proteins A (MICA) and major histocompatibility complex class I molecular-associated proteins B (MICB) are highly expressed on the various tumor cells membrane, acting as ligands of natural killer group 2 member D (NKG2D) expressed on NK cells and γδT cells. NKG2D interacts with its ligand for immune surveillance and lysis of tumor cells, and this process is regulated by HIF-1α [80, 81]. In pancreatic cancer, HIF-1α and ADAM10, a disintegrin and metalloproteinase domain 10 (ADAM10) are highly expressed and are negatively regulated by miR-153. Overexpressed circ-0000977 serves as a sponge for miR-153 to counteract miR-153-mediated suppression of HIF-1α and ADAM10, promoting the shedding of membrane MICA (mMICA) from surface of tumor cells and converting into soluble MICA (sMICA). SMICA binds to NKG2D, down-regulating NKG2D expression as well as cytotoxicity of NK cells, resulting in immune evasion [82].

#### Desmoplasia

Pancreatic cancer is characterized by desmoplasia and highly hypoxic microenvironment composed of tumor cells, extracellular matrix, fibroblasts, endothelial cells and immune cells, the both amplify each other in a positive feed-back loop and accelerate pancreatic cancer progression [83, 84]. In hypoxic microenvironment, hypoxia-induced chemokines attract monocytes/macrophages to inflammation, damage tissues and tumor tissues. In pancreatic cancer, hypoxia-induced HIF-1α bound to the HRE of chemical chemokine 2 (CCL2) and increased its expression, recruiting macrophages to infiltrate tumor tissues to activate pancreatic stellate cells and confer it fibroblast phenotypes via increasing the expression of α-smooth muscle actin, aggravating the hypoxic microenvironment of pancreatic cancer and accelerating its progression [85]. Studies indicated sonic hedgehog (SHH) ligand was conducive to occurrence of desmoplasia, and paracrine hedgehog (HH) signaling played a central role in tumorigenic communication between tumor cells and fibroblasts in the stroma in pancreatic cancer. Hypoxia increased the expression of SHH in a HIF-1α-dependent manner, and its overexpression activated HH signaling and collagen Ι fibronectin formation, inducing desmoplasia and enhancing tumor aggressiveness in pancreatic cancer [84, 86].

#### Prospective into clinical application

HIF-1α manipulates the malignant biological features of pancreatic cancer through various pathways. Hence, targeting HIF-1α and its signaling pathways could be potential therapeutics for pancreatic cancer. It is difficult to block HIF-1α directly because it is a transcription factor and mainly locates in the nucleus [5]. Currently, promoting the degradation of HIF-1α protein and targeting certain molecules in the HIF-1α signaling pathways are effective therapeutics for pancreatic cancer. As is known to all, HIF-1α is degraded via the ubiquitin-mediated proteasome-dependent pathway under normoxia and is stable in hypoxia. Nevertheless, infection of pancreatic cancer cells with oncotropic H-1 parvovirus could rapidly degrade HIF-1α in a proteasome-dependent manner in hypoxia, and this process was independent of Von Hippel–Lindau and receptor of activated protein C kinase [87]. Analogously, pharmacologic ascorbate (P-AscH) rapidly degrades HIF-1α in a proteasome-dependent pathway in pancreatic cancer cells which is independent of 2-oxoglutarate-dependent prolyl hydroxylase (PHD-2). P-AscH can also increase extracellular H_2_O_2_ and transport it into tumor cells through the plasma membrane to inhibit the expression of HIF-1α and VEGF, exerting a killing effect on pancreatic cancer cells [88]. Besides, extracellular superoxide dismutase could accelerate the degradation of HIF-1α via reducing peroxides in pancreatic cancer [89]. The study showed xylene derivative TEL03 could bind to HIF-1α to block the combination of HIF-1α and p300, and induce the degradation of HIF-1α by proteasome pathway in pancreatic cancer. Moreover, TEL03 could inhibit the phosphorylation of STAT3, acting as an upstream molecule and transcriptional factor of HIF-1α, suppressing HIF-1α expression [90]. Notably, TX-2098, a hypoxia cytotoxin, directly down-regulated the protein level of HIF-1α and its downstream targets such as VEGF, glucose transporter type 1 and aldolase A in pancreatic cancer, inhibiting its progression [91]. Prolyl hydroxylase domain 3 (PHD3), a rate-limiting enzyme regulating HIF-1α degradation, improves radiotherapy efficacy through inhibiting p-EGFR/HIF-1α signaling in pancreatic cancer [92].

In addition to the acceleration of HIF-1α degradation, targeting the HIF-1α signaling pathway is also a momentous avenue to impede the progression of pancreatic cancer. Alpha-solanine, a steroidal alkaloid with anti-tumor effects extracted from plants of the family Solanaceae, inhibits pancreatic cancer cells proliferation, migration and invasion through targeting p-ERK1/2-HIF-1α-VEGF axis [93, 94]. Similarly, HS-345, an inhibitor targeting tropomyosin-related kinase A, and HS-527, an inhibitor of PI3K, inhibit angiogenesis via targeting HIF-1α/VEGF axis in pancreatic cancer [95, 96]. Besides, curcumin, a natural polyphenol present in turmeric, and its analogues suppress the expression of HIF-1α by inhibiting its interaction with HSP90 and NF-κB signaling pathway in pancreatic cancer [97]. Triptolidenol-1 (LB-1) is a derivative of LB, with less toxicity. In pancreatic cancer, LB-1 decreased the activity of HIF-1α via inhibiting its upstream pathway PI3K/Akt/mTOR, moreover, LB-1 could inhibit the connection between HIF-1α, p-STAT3 and p300, repressing VEGF expression, in addition, LB-1 accelerated HIF-1α degradation by ubiquitin–proteasome pathway [98]. Additionally, in pancreatic cancer, combination of PX-478, a specific agent suppressing constitutive and hypoxia-induced expression of HIF-1α, with gemcitabine induce immunogenic cell death which is related to repression of HIF-1α via upregulating phosphorylation of eIF2α [99], PX-478 can enhance radiosensitization by inhibition of HIF-1α as well [100].

Notable is, studies indicate that two types of hypoxia are present in tumor, chronic and cycling hypoxia. Cycling hypoxia more appropriately describes the dynamic changes of hypoxia and reoxygenation in tumor and has been demonstrated to induce tumor aggressiveness more significantly than chronic hypoxia [101–103]. At present, the hypoxia status in most studies is set to chronic hypoxia, and the duration of hypoxia and the oxygen concentration are not consistent among the studies, which cannot completely simulate the pancreatic cancer hypoxic microenvironment in vivo, causing experimental results instability potentially. Besides, each study selects specific pancreatic cancer cell lines, and the molecular mechanisms proven in these studies might not be applied to all pancreatic cancer cells, which limit their application to the clinical treatment of pancreatic cancer possibly. HIF-1α is in a complex signaling network and regulates the biological characteristics of pancreatic cancer cells in diverse manners. Therefore, future studies should focus on the entire signal network and explore central regulatory molecules to effectively inhibit pancreatic cancer progression. Our manuscript integrates acting mechanisms of HIF-1α to provide a comprehensive perspective of the role of HIF-1α in pancreatic cancer, showing theoretical principle for the targeted therapy of pancreatic cancer.

## Conclusions

**Fig. 3:**
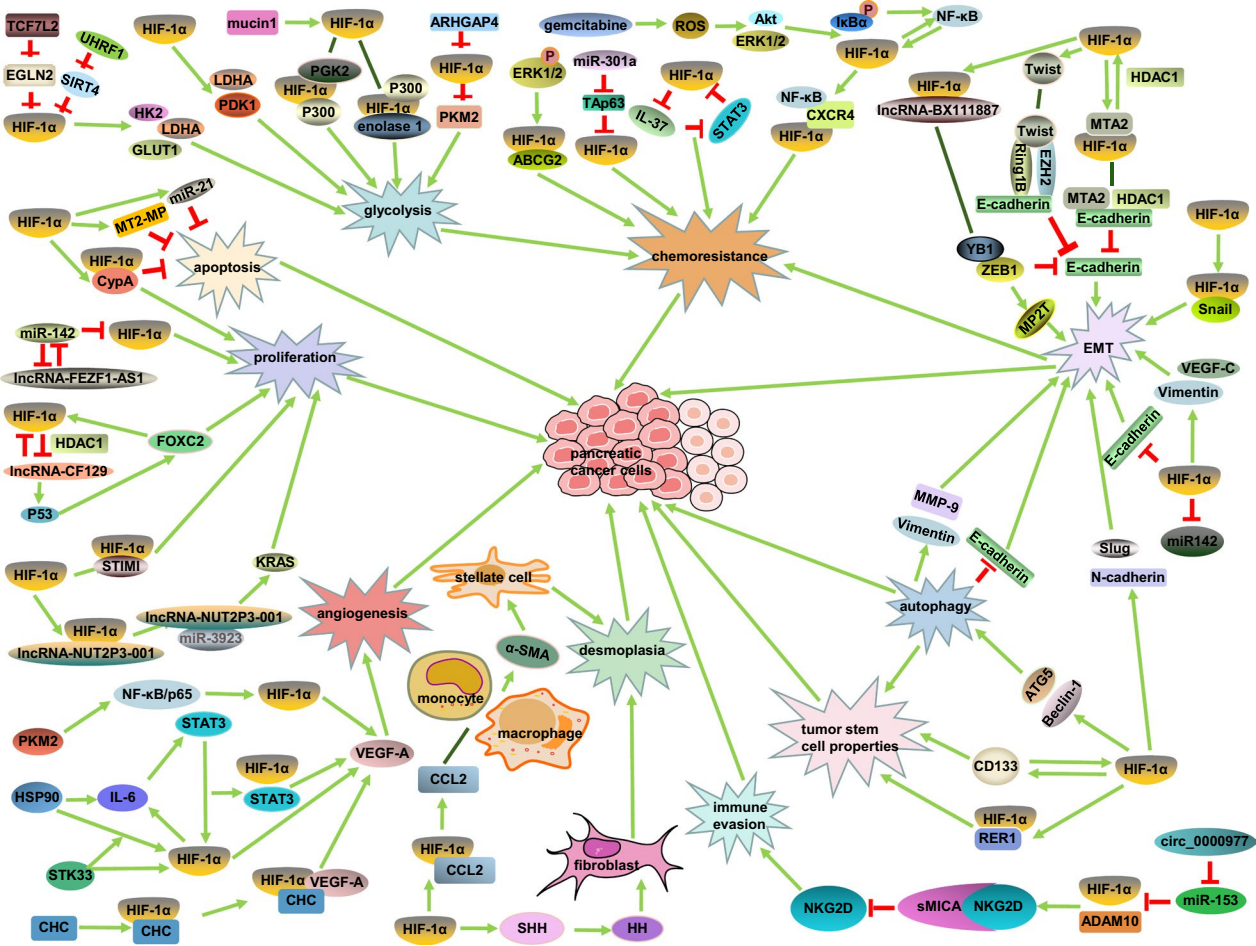
Signaling pathways induced by HIF-1α in pancreatic cancer tumorigenesis and progression. ↑, promote; Τ, inhibit; ׀, recruit

As shown in Fig. 3 in pancreatic cancer hypoxic microenvironment, HIF-1α induces tumor cells malignant biological characteristics and mediates tumorigenic crosstalk between tumor parenchyma and stroma. This suggests novel therapeutic strategies targeting HIF-1α and  its signaling pathways might be promising for pancreatic cancer therapy.

## Data Availability

All data are included in this article.

## Abbreviations

HIF-1: Hypoxia-inducible factor-1
HIF-1α: Hypoxia-inducible factor-1alpha subunit
HIF-1β: Hypoxia-inducible factor-1 beta subunit
CBP: Cyclic adenosine monophosphate response element-binding protein
p300: Acetyltransferase
HRE: Hypoxia response element
ARNT: Aryl hydrocarbon receptor nuclear translocator
VEGF-A: Vascular endothelial growth factor A
KRAS: V-Ki-ras2 Kirsten Rat Sarcoma Viral Oncogene Homolog
HDAC1: Histone deacetylase 1
FOXC2: Forkhead box C2
STIM1: Stromal interaction molecule 1
ARHGAP4: Rho GTPase-activating protein 4
PKM2: M2 isoform of pyruvate kinase
ENO1: Enolase1
PGM2: Phosphoglucomutase-2
TCF7L2: Transcription factor 7-like 2
EGLN2: Egl-9 family hypoxia-inducible factor 2
CypA: Cyclophilin A
MT2-MMP: Membrane type-2 matrix metalloproteinase
EMT: Epithelial mesenchymal transition
E-cadherin: Epithelial cell cadherin
MTA2: Metastasis-associated protein 2
EZH2: Enhancer of zeste homolog 2
ZEB1: Zinc finger E-box-binding protein 1
RER1: Retention in endoplasmic reticulum 1
ABCG2: ATP-binding cassette subfamily G member 2
CXCR4: Chemokine receptor 4
CXCL12: Chemokines 12
STAT3: Signal transducer and activator of transcription 3
HSP90: Heat shock protein90
STK33: Serine/threonine kinase 33
CHC: Clathrin heavy chain
MMP-2: Matrix metalloproteinase 2
MMP-9: Matrix metalloproteinase 9
MICA: Major histocompatibility complex class I molecular-associated proteins A
MICB: Major histocompatibility complex class I molecular-associated proteins B
NKG2D: Natural killer group 2 member D
ADAM10: A disintegrin and metalloproteinase domain 10
sMICA: Soluble MCIA
sMICB: Soluble MCIB
CCL2: Chemical chemokine 2
SHH: Sonic hedgehog
HH: Hedgehog
P-AscH: Pharmacologic ascorbate
PHD-2: 2-oxoglutarate-dependent prolyl hydroxylase
PHD3: Prolyl hydroxylase domain 3
LB-1: Triptolidenol-1
